# Should glucagon‐like peptide‐1 receptor agonist treatment be withheld in preoperative management?

**DOI:** 10.1111/1753-0407.13469

**Published:** 2023-09-19

**Authors:** Zachary Bloomgarden

**Affiliations:** ^1^ Department of Medicine, Division of Endocrinology, Diabetes, and Bone Disease Icahn School of Medicine at Mount Sinai New York NY USA

The American Society of Anesthesiologists recently released a consensus statement suggesting that patients treated with weekly glucagon‐like peptide‐1 receptor agonists (GLP‐1RA) have delayed gastric emptying and should therefore be instructed not to take these agents for 1 week before an elective procedure that might be associated with risk of regurgitation and pulmonary aspiration of gastric contents.^1^ The authors of the statement acknowledged that the evidence for the recommendation “is sparse limited only to several case reports.”^1^

Is this a reasonable recommendation?

Delayed gastric emptying is a recognized effect of GLP‐1RA^2^ and not only contributes to greater satiety but also is likely to be a mechanism underlying the reduction in postprandial glycemia with these agents. The normal half‐time of gastric emptying is approximately 2 h,^3^ so that an important question is whether the ~30% delay in gastric emptying reported with GLP‐1RA such as semaglutide at 4 h^4^ is likely to lead retention of gastric contents after the 8–12 h period of fasting typically advised for persons undergoing surgery and other procedures associated with risk of pulmonary aspiration. GLP‐1RA are hardly unique in delaying gastric emptying. A large number of medications potentially interfere with gastric motility, including narcotic analgesics, anticholinergics, antidepressants, calcium channel blockers, and gastric acid suppressants^2^; anesthesiologists do not generally recommend that such medications be omitted by persons undergoing surgery. Certainly, the stated goal of the consensus statement to avoid the “potential risk of regurgitation and pulmonary aspiration of gastric contents”^1^ is laudable, but we wonder whether an important class of drugs recognized to be associated with gastrointestinal side effects during the initial 8–12 weeks of administration is being unreasonably singled out.

Are there potential adverse effects of withholding GLP‐1RA in the perioperative period? There is extensive evidence that these agents are potent in lowering glycemia and indeed have glucose‐lowering benefit similar to that of basal insulin, without causing hypoglycemia or weight gain.^5^ If GLP‐1RA are discontinued 1 week or more before a surgical procedure, alternatives are to administer supplemental insulin, increasing the likelihood of hypoglycemia, and perhaps increasing cardiovascular disease (CVD) risk,^6^ or to accept higher glucose levels in the perioperative period, with attendant increases in likelihood of wound infection and mortality.^7^, ^8^ An even more worrisome consideration stems from the evidence of cardiovascular outcome benefits of GLP‐1RA seen over the long term^9^; the question must be asked as to the safety of withholding these cardioprotective agents during the perioperative period, which is recognized to be associated with particularly high CVD risk.^10^

We must consider the injunction, *primum non nocere*, and recognize that by withholding a well‐tolerated treatment that is playing an important role in the management of a persons with diabetes we may actually create a “potential for harm.^11^”
